# Clinical characteristics and time trends of hospitalized methadone exposures in the United States based on the Toxicology Investigators Consortium (ToxIC) case registry: 2010–2017

**DOI:** 10.1186/s40360-020-00435-0

**Published:** 2020-07-22

**Authors:** Omid Mehrpour, Christopher Hoyte, Alireza Amirabadizadeh, Jeffrey Brent, On behalf of the Toxicology Investigators Consortium (ToxIC)

**Affiliations:** 1 Rocky Mountain Poison and Drug Center, Denver Health and Hospital Authority, 1391 Speer Blvd, 777 Bannock St. MC 0180, Denver, CO 80204 USA; 2 Medical Toxicology and Drug Abuse Research Center (MTDRC), Birjand University of of Medical Sciences, Birjand, Iran; 3 Department of Emergency Medicine and Medical Toxicology, University of Colorado Anschutz Medical Campus, University Hospital, Aurora, CO USA; 4 Cardiovascular Diseases Research Center Birjand University of Medical Sciences, Birjand, Iran; 5 School of medicine, University of Colorado, Aurora, CO USA

**Keywords:** Methadone, Poisoning, Trend, Toxicology investigators consortium (ToxIC), United States

## Abstract

**Background:**

Methadone is well known for its long duration of action and propensity for mortality after an overdose. The present research was aimed at evaluating the clinical manifestations and time trends of methadone exposure in patients in US hospitals.

**Methods:**

We queried the American College of Medical Toxicology’s Toxicology Investigators Consortium case registry for all cases of methadone exposure between January 1, 2010, and December 31, 2017. The collected information included demographic features, clinical presentations, therapeutic interventions, poisoning type (acute, chronic, or acute on chronic), and the reason(s) for exposure. Descriptive data and relative frequencies were used to investigate the participants’ characteristics.

Our data analysis was performed using SPSS version 19 and Prism software. The trends and clinical manifestations of methadone poisoning over the time period of the study were specifically investigated.

**Results:**

Nine hundred and seventy-three patients who met our inclusion criteria, with a mean age of 41.9 ± 16.6 years (range: 11 months-78 years) were analyzed. Five hundred eighty-two (60.2%) were male. The highest rate of methadone poisoning was observed in 2013. There was an increasing rate of methadone exposures in 2010–2013, followed by a decline in 2014–2017. The most common clinical manifestations in methadone-poisoned patients were coma (48.6%) and respiratory depression (33.6%). The in-hospital mortality rate of methadone poisoning was 1.4%.

**Conclusion:**

ToxIC Registry data showed that inpatient methadone exposures enhanced from 2010 to 2013, after which a reduction occurred in the years 2014 to 2017.

## Background

Methadone was developed in Germany in 1937 and introduced to the United States (US) in 1947 [1]. It is a prescription opioid and is currently one of the primary options for the medication-assisted therapy of opioid use disorder [2] and for alleviating chronic pain. However, it has a high abuse potential [3]. Methadone has the highest rate of mortality in overdose of prescribed opioid analgesics in the US [4] and is an important cause of opioid-related deaths in many other countries [5]. In 2011, in the US alone, approximately 180,000 patients were reported to be on methadone maintenance therapy [6]. Another survey in the US reported that the number of individuals receiving methadone increased from about 227,000 in 2003 to over 350,000 in 2015 [7].

Along with the increased use of methadone comes an increased risk of side effects or adverse reactions related to overdose, such as rhabdomyolysis, sedation, sweating, respiratory depression, dizziness, nausea, dysrhythmias, vomiting, itching, constipation, orthostatic hypotension, prolongation of the QT interval, and death [1]. Despite the large numbers of potentially serious sequelae of methadone use, few studies have evaluated trends in methadone toxicity in the US. Therefore, we aimed to evaluate the trends and patient-related factors associated with cases of methadone exposure seen in US hospitals by utilizing a well-established prospective clinical database.

## Methods

We queried the Toxicology Investigators Consortium (ToxIC) Case Registry for all cases of methadone poisoning recorded between Jan 1, 2010, and December 31, 2017. The ToxIC Registry prospectively records cases cared for by participating medical toxicologists. It was designed to collect data by medical toxicologists and thus is felt to represent toxicologically accurate information. Members of the Consortium consist of all medical toxicologists from participating sites. Although this has varied from year to year, there are currently 49 sites participating in ToxIC, comprising the majority of US medical toxicology training programs and practices.

Patient data is entered into the ToxIC Registry via an online interface on which information is recorded on the substances involved, patient demographics, presenting signs and symptoms, toxidromes, treatments administered, and outcomes. The ToxIC Registry has been described in detail previously [8–10].

For the current study, we queried the ToxIC Registry for the following patient variables: gender, age, race, data pertaining to the cause of the exposure, agents involved, route of exposure (e.g. oral, parenteral), clinical manifestations, including toxidromes, abnormalities of vital signs, renal, cardiovascular, nervous system, gastrointestinal, metabolic, pulmonary, hematologic, muscle, and dermatologic effects; therapeutic interventions, including antidotes, medication treatment, decontamination, elimination techniques, and pharmacologic and non-pharmacologic support. Intentional methadone exposure was defined as any ingestion taken for therapeutic purposes, self-harm, or misuse/abuse.

Our inclusion criterion was any inpatient case in the ToxIC Registry where methadone was an implicated causal agent. Patients were excluded if the data related to age, sex, or cause of toxicological consultation was missing, if they were outpatients, or if they were seen for methadone withdrawal. Accidental ingestions, and those due to pediatric exploratory behavior, were classified as unintentional. All other cases were classified as intentional exposures. The analyses included all patients of any age meeting inclusion criteria who were registered during the study period.

The ToxIC project took place after review by the Western IRB and individual IRBs of ToxIC sites. All data in the ToxIC Registry is patient-deidentified and collected during routine clinical care. It does not involve any patient interventions.

### Statistical and analytical methods

Descriptive statistics and relative frequencies plus graphical techniques were applied for investigating the patients’ features. Data analysis was performed using SPSS version 19 and Prism software. Descriptive data, including frequency, percentage, mean, and standard deviation, were extracted and analyzed. Variables are reported as mean or median ± standard deviations.

Using the Chi-square test, we investigated the frequency distribution of clinical manifestations (coma, respiratory depression, seizure, etc.) in single- and co-exposure cases. Also, using this test, we examined and compared the frequency distribution of administered treatment patients with single- and co-exposures. For comparing the mean methadone dose consumed, after investigating the normality using the Kolmogorov-Smirnov test through the Mann-Whitney nonparametric test, we compared the methadone doses, QTc, and the effect rates in patients with single- or co-exposure to methadone. Also, the frequency distribution of clinical effects was reported based chronicity of use in all methadone patients.

Distribution of the total number of intentional and unintentional methadone poisoning cases reported to the ToxIC Registry 2010–2017 was assessed by the Chi-square test. *P* values of smaller than 0.05 were regarded as significant, although data were presented without regard to formal statistical significance.

## Results

Nine hundred and seventy-three patients who met our inclusion criteria, with a mean age of 41.9 ± 16.6 years (range: 11 months-78 years), and a median age of 45.0 years were analyzed. Seven cases were excluded based on our exclusion criteria (6 were outpatients, and one patient had missing data). Eight hundred and thirty-one (86.0%) patients were 19–65 years old, and 28 (2.9%) were under the age of 2 years. Five hundred eighty-two (60.2%) were male.

Three hundred and fifty-four cases (36.4%) had methadone-only exposures, and 619 (63.6%) had had co-ingestants (Table 2). The mean dose of methadone in all cases was 111 ± 122 mg (mg) (range: 3–800), with a median dose of 90 mg. In the methadone-only group, the mean methadone dose was 114 ± 129, with a median of 95 mg. For the group with co-ingestants, the mean methadone dose was104 ± 124, with a median of 80 mg. The mean methadone dose in patients who received naloxone was 112 ± 108, with a median of 91 mg. In patients who did not receive naloxone, the mean methadone dose was 110 ± 137, with a median of 90 mg. The Mann-Whitney test did not show any significant difference in the methadone dose between groups that did or did not receive naloxone (*p* = 0.18).

The route of exposure was known in 437 (44.9%) patients. Of these, 420 (44.8%) patients consumed methadone orally, and 17 (1.8%) used a parenteral route. The chronicity of exposure, known in 607 patients, was acute in 411 (67.7%) patients, acute on chronic in 136 (14.5%), and chronic in 60 (6.4%) patients.

Six hundred and fifty-two (67.6%) patients had been referred to the medical toxicology service by the emergency department, 120 (12.5%) by the admitting service, 86 (8.9%) by another hospital service, and 66 (6.8%) were transferred from outside hospitals (Table 1). The in-hospital mortality rate of methadone poisoning was 1.4% (14 patients).

**Table 1:** Frequency of sources of referral to medical toxicology services and demographic information

Variable	Frequency /mean	Percent /SD
**Age (**year)	41.9	16.6
**Dose** (milligram)	111.34	121.78
**Source of referral**		
Emergency department (ED)	652	67.5
Admitting Service	120	12.4
Outside Hospital Transfer	66	6.8
Poison center	12	1.2
Request from another hospital service	86	8.9
PCP or other Outpatient Treating MD	14	1.4
Self-Referral	1	0.1
Unknown	15	1.5
**Gender**		
Male	584	60.0
Female	389	40.0
**Role of medical toxicologist and location of toxicology consultation**		
Attending (Inpatient)	179	19.9
Consult (ED/Inpatient)	757	80.1
**Chronicity of exposure**		
Acute	411	67.7
Acute on chronic	136	14.5
Chronic	60	6.4
**Reason for methadone use**		
Withdrawal management	51	5.2
Abuse	209	21.4
Attempt at self-harm	508	52.2

**Fig. 1:**
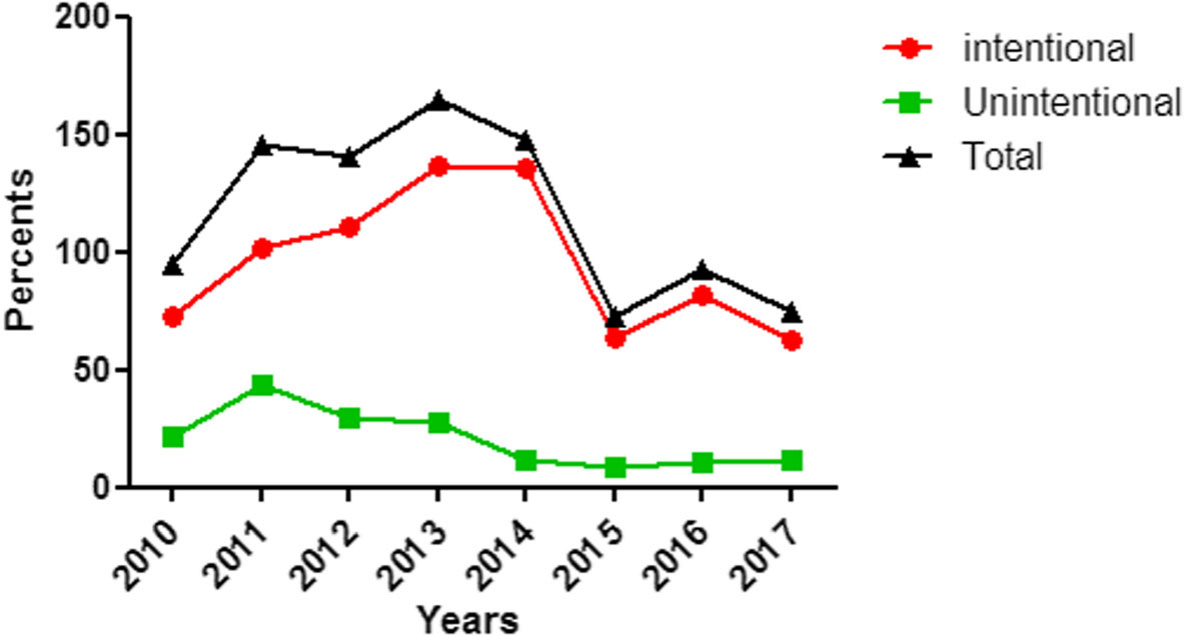
Distribution of the total number of methadone poisoning cases reported to the ToxIC Registry 2010–2017

Seven hundred and sixty-eight (79.5%) patients had intentional methadone exposures. Of these, 51 (6.6%), 209 (27.3%), and 508 (66.1%) were due to avoidance of withdrawal, drug abuse, and attempts at self-harm, respectively. As shown in Fig. 1, the highest rate of methadone poisoning was observed in 2013. There was an increasing rate of methadone exposures in 2010–2013, followed by a decline in 2014–2017. The highest number of intentional methadone poisoning cases was clearly increasing in 2010 and peaked in 2013–2014 with 137 and 136 cases, respectively. After 2014, there was a decline. The highest frequency of unintentional methadone poisoning was reported in 2011 (44 patients).

**Fig. 2:**
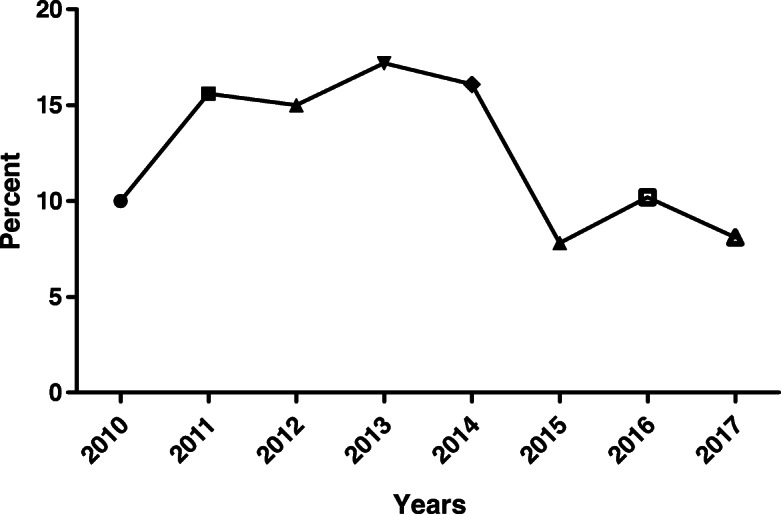
The percent of all methadone cases/total cases to the ToxIC Registry by year. Frequency distribution of methadone poisoning cases in 2015 had a significant difference with years of 2011, 2012, 2013, 2014 (*p* < 0.001). Frequency distribution of methadone poisoning cases in 2017 had a significant difference with years of 2011, 2012, 2013, 2014 (*p* < 0.001). Frequency distribution of methadone poisoning cases in the 2010 as well as 2016 had a significant difference with years of 2011, 2012, 2013, 2014 (*p* < 0.001).

Chi-square testing comparing the different years during our study period indicated that the causes of methadone toxicity significantly varied with time (X2 = 295.81, *p* < 0.001). The highest percent of methadone cases reported to the ToxIC Registry relative to the overall number of cases reported to the Registry for each year was obtained in 2013 (n: 169 [17.5% of all methadone cases reported]), and the lowest rate was in 2015 (n: 77 [7.9%] [Fig. 2]). As is evident in Fig. 2, the frequency distribution of methadone poisoning cases in 2015 had a significant difference from the years 2011, 2012, 2013, 2014 (*p* < 0.001). The frequency distribution of methadone poisoning cases in 2017 had a significant difference from the years of 2011, 2012, 2013, 2014 (*p* < 0.001). Frequency distribution of methadone poisoning cases in 2010, as well as 2016, had a significant difference from the years of 2011, 2012, 2013, 2014 (*p* < 0.001). Tables 2 and 3 show the distribution of clinical effects and mean doses associated with various clinical manifestations for single-agent and co-ingestant exposure to methadone. In patients with co-ingestants, 88 (14.3%) co-ingested sedative-hypnotics, 35 (3.6%) oxycodone, 27 (2.8%) heroin, and 353 (34.0%) other substances.

**Table 2:** The percent of clinical effects in methadone alone poisoning and co-ingestion

Variable	Total	Single exposure of methadone (*n* = 354)	Co exposure of Methadone with other drugs (*n* = 619)
Coma/CNS	472 (48.6%)	354 (100%)	118 (19.1%)
Agitation	69 (7.1%)	39 (11.1%)	30 (4.84%)
Seizures	21 (2.2%)	13 (3.67%)	8 (1.29%)
Weakness	6 (0.6%)	2 (0.56%)	4 (0.64%)
pH < 7	57 (5.8%)	43 (12/0%)	14 (2.26%)
Bradycardia	41 (4.2%)	26 (7.28%)	15 (2.42%)
Hypertension	34 (3.5%)	24 (6.72%)	10 (16.1%)
Hypotension	38 (3.9%)	28 (7.91%)	10 (16.1%)
Tachycardia	22 (2.3%)	13 (3.67%)	9 (1.45%)
QTc > 500 milliseconds	24 (2.9%)	20 (5.64%)	4 (0.64%)
Acute Kidney injury	92 (9.5%)	70 (19.7%)	22 (3.55%)
Rhabdomyolysis	43 (4.4%)	35 (9.88%)	8 (1.29%)
Aspiration pneumonitis	53 (5.4%)	44 (12.4%)	9 (1.45%)
Respiratory depression	327 (33.6%)	251 (70.9%)	76 (12.3%)
Hepatotoxicity (AST > 1000)	36 (3.7%)	28 (7.91%)	8 (1.29%)

Values are frequency and percentage

The percentages in the methadone only and methadone plus coingestants are calculated in each group

*Abbreviations*: *AST* aspartate aminotransferase, *CNS* central nervous system

**Table 3:** Comparison of methadone doses and effect rates in patients with single or co-ingestant exposure of methadone

Complications		Dose of methadone in methadone alone (mg)	Dose of methadone in methadone plus other drugs (mg)
Coma/Central nervous system (CNS) depression	Yes	96.2 [56.8–125.0]	97.2 [72.3–136.0]
	No	65.9 [38.6–84.1]	124.3 [100.1–168.7]
Agitation	Yes	132.5 [98.3–140.7]	119.3 [108.9–127.1]
	No	125.0 [96.2–156.4]	37.5 [29.8–65.3]
	No	66.6 [54.8–89.2]	121.3 [95.6–163.8]
Seizures	Yes	138.2 [96.5–165.1]	136.9 [103.6–172.3]
	No	145.2 [100.2–189.0]	120.1 [89.6–160.3]
Weakness	Yes	43.7 [35.6–57.6]	43.6 [32.1–68.9]
	No	14.2 [9.6–25.6]	119.6 [100.0–156.3]
pH < 7	Yes	40.2 [24.3–50.8]	45 ± 24.2
	No	69.8 [57.3–99.2]	124.3 [100.6–154.9]
Bradycardia	Yes	42.3 [36.8–60.2]	64.5 [49.6–87.0]
	No	38.6 [30.1–45.7]	119.9 [96.3–157.6]
Hypertension	Yes	40.2 [32.3–50.9]	126.3 [100.5–160.0]
	No	39.6 [28.9–61.2]	120.3 [95.6–156.8]
Hypotension	Yes	32.0 [25.3–41.9]	26.3 [14.6–38.9]
	No	40.9 [32.9–53.6]	126.5 [105.3–153.6]
Tachycardia	Yes	74.9 [62.3–90.4]	147.1 [125.3–175.1]
	No	36.0 [28.9–51.4]	123.6 [98.0–168.6]
QTc > 500 milliseconds	Yes	74.2 [52.8–88.6]	32.1 [29.6–36.5]
	No	34.5 [28.6–58.9]	128.7 [106.5–150.6]
Acute Kidney injury	Yes	42.5 [14.8–65.3]	16.8 [9.8–26.1]
	No	117.3 [84.0–140.4]	102.3 [86.7–123.9]
Rhabdomyolysis	Yes	37.5 [29.7–50.0]	42.3 [19.0–57.8]
	No	43.8 [34.6–59.9]	124.3 [97.8–150.1]
Aspiration pneumonitis	Yes	24.6 [16.7–36.8]	37.8 [26.3–49.0]
	No	28.6 [14.6–34.5]	124.8 [100.1–148.6]
Respiratory depression	Yes	80.3 [60.3–100.2]	46.6 [34.8–67.2]
	No	128.7 [100.2–168.7]	108.9 [85.3–125.6]
Hepatotoxicity (AST > 1000)	Yes	45.2 [25.6–59.9]	43.2 [29.7–60.0]
	No	62.4 [48.7–81.0]	122.3 [99.3–145.6]

Dose values are mean ± Standard deviation

*: Mann-Whitney test

z = test statistics Mann-Whitney

The median (25th%-75th%) QTc in patients with methadone poisoning was 446.0 [430.0–480.0] milliseconds. The median (25th%-75th%) QTc in single-exposure and co-exposure methadone patients were 449.9 [438.0–462.9] and 435.2 [389.9–480.0] milliseconds, respectively (*p* = 0.28).

The most common clinical manifestations in methadone poisoned patients were coma (48.6%) and respiratory depression (33.6%) (Table 2), occurring at mean doses of 96.2 [56.8–125.0] and 37.5 [29.7–50.0] mg, respectively. Two percent of patients experienced seizures. The median dose of methadone in patients with seizures who ingested methadone alone was 138.2 [96.5–165.1] mg, similar to the dose of methadone in patients with seizures in the co-ingestion group, which was 136.9 [103.6–172.3] mg (Table 3). The median (25th%-75th %) QTc in patients with and without seizure was 453.0 [438.0–481.0] and 441.0 [401.0–495.0] milliseconds respectively (*p* = 0.49).

Table 4 shows the frequency of treatments, stratified by single and polydrug methadone poisoning cases. Among all patients, naloxone was the most commonly used antidote. Four hundred and forty-two patients (45.4%) received naloxone. Benzodiazepines, the second most frequently administered class of agents, were given to 119 (12.3%) patients. Of those receiving benzodiazepines, 20 patients had seizures, 32 patients had agitation, 46 had withdrawal, and the reason for receiving benzodiazepine in 21 patients was unknown.

**Table 4:** Comparison of administered treatments in patients with single and co-ingestant exposures to methadone

Variable		Total	Single exposure of methadone (*n* = 354)	Co exposure of Methadone with other drugs (*n* = 619)
Naloxone	Yes	442 (45.4%)	125 (35.3%)	317 (51.2%)
	No	531 (54.6%)	232 (65.5%)	299 (48.3%)
N-acetylcysteine	Yes	40 (4.2%)	27 (7.6%)	13 (2.1%)
	No	933 (95.8%)	330 (93.2%)	603 (97.4%)
Flumazenil	Yes	22 (2.3%)	9 (2.5%)	13 (2.1%)
	No	951 (97.7%)	348 (98.3%)	603 (97.4%)
Sodium bicarbonate	Yes	21 (2.2%)	13 (2.77%)	8 (1.29%)
	No	952 (97.8%)	344 (97.2%)	608 (98.2%)
Vasopressors	Yes	34 (3.5%)	26 (7.34%)	8 (1.29%)
	No	939 (96.5%)	331 (93.5%)	608 (98.2%)
Anticonvulsants	Yes	13 (1.4%)	8 (2.3%)	5 (0.81%)
	No	960 (98.6%)	349 (98.6%)	611 (98.7%)
Antipsychotics	Yes	25 (2.6%)	10 (2.8%)	15 (2.42%)
	No	948 (97.4%)	347 (98.2%)	601 (97.1%)
Benzodiazepine	Yes	119 (12.3%)	66 (18.6%)	53 (8.56%)
	No	854 (87.7%)	291 (81.5%)	563 (90.9%)
Opioids	Yes	38 (4.0%)	27 (8.22%)	11 (1.77%)
	No	935 (96.0%)	330 (93.2%)	605 (97.7%)

Sixty-six percent of patients who experienced coma had acute poisoning, while 47.8% had acute on chronic poisoning. Of patients who experienced respiratory depression, 49.4% had acute poisoning, while 66.9% had acute on chronic poisoning, and 5% had chronic poisoning. (Table 1 in supplemental data). Three-hundred and eight (69.8%) patients with coma and 226 (51.3%) patients with respiratory depression received naloxone (Table 2 in supplemental data).

## Discussion

Methadone has been used therapeutically to alleviate pain in patients with chronic disease and to reduce and control withdrawal syndrome in patients who suffer opioid dependency syndrome in methadone maintenance treatment (MMT) clinics [11]. It has a high potential for abuse and may be used illicitly by opioid-dependent patients [11].

Methadone is well known for its long duration of action and potential for fatality in overdose. This places significant health care and economic burdens on society, especially where death occurs. Mortality costs attributed to methadone accounted for approximately 6.5 million dollars in 2009 in the US [12]. Other studies showed that patients who receive methadone to alleviate chronic pain in pain clinics are at higher risk of mortality [11]. Patients who have chronic pain tend to be older individuals in poorer health who may be receiving multiple medications and experiencing high levels of depression and anxiety. Methadone may be abused by individuals with opioid misuse disorder, which increases its risks [11]. Our study demonstrated that rates of methadone toxicity in the US, as reflected in the ToxIC database, appeared to increase until 2013–2014, after which there was a decline. Since the ToxIC database is a reflection of cases for which medical toxicology consultation was required, it is likely that more trivial cases are not included. Thus, these data should be interpreted as reflecting significant poisonings.

Another study with different study period has shown that hospital discharge frequency for methadone poisoning rose dramatically through 1997–2007, and then significantly declined through 2007–2014 [13]. This discrepancy may be due to the different study periods and study populations. In that study, the authors analyzed national trends in inpatient and emergency department discharges for opioid abuse, dependence, and poisoning, but in this study, we analyzed just inpatient methadone poisoning cases. In 2005, the Researched Abuse, Diversion and Addiction-Related Surveillance (RADARS) System reported that there was a correlation between the increasing trend in methadone prescriptions and the degree of diversion and abuse, with no meaningful difference in the number of people on methadone maintenance therapy [14]. More recently, the number prescriptions for all opioids has decreased, associated with awareness by practitioners of the dangers of opioids, and national, state, and local measures for reducing the prescribing of opioids [13, 15–17].

The US Centers for Disease Control and Prevention reported that prescriptions for opioids peaked in 2012, with greater than 255 million filled (81.3 prescriptions for every 100 persons). The total national opioid prescription frequency then decreased between 2012 and 2017, with the lowest rate in the last ten years in 2017, at 58.7 prescriptions for every 100 persons. However, this still represents more than 191 million opioid prescriptions filled [18]. This is consistent with our data. We showed a peak in methadone poisoning in 2013 and 2014, and after that, there was a decline in the number of methadone poisoning cases.

In our study, the mortality rate due to methadone poisoning was 1.4%. However, our patients were admitted to hospitals and thus were alive at presentation. It is possible, however, that the rate of death from methadone poisoning in cases that do not reach a hospital is much higher [19].

Methadone abuse is an important ongoing epidemic, and the 2017 data from the National Poisoning Data System illustratively contains 1054 single methadone poisonings, 456 intentional methadone overdoses, and 56 deaths associated with methadone [20]. Similarly, in a study by Dart et al., methadone was the leading opioid as a cause of death in the NPDS, with 178 cases in 2012 [4].

Coma and respiratory depression were the most common signs of severe methadone toxicity in our study. In a cohort of prescription opioid overdose patients, methadone was the second most commonly prescribed opioid after oxycodone. They showed that the risk factors for severe respiratory depression in patients with prescription opioid overdose include drug misuse (pertinent for methadone), increased age, and the specific opioid medication involved. In that study, methadone had a much higher risk of severe respiratory depression [21]. Other studies revealed that a history of a substance use disorder was closely associated with the development of opioid-induced respiratory depression, with an odds ratio of 12.7 [22].

Patients experiencing these complications ingested a mean of 104 mg, which is a lower average dose than our entire cohort. This is likely because non-opioid-tolerant patients are the most vulnerable to adverse effects of opioids, even at lower doses, and there was an over-representation of acute ingestions in the group with coma and respiratory depression. Almost 3% of patients had QTc prolongation, a known, yet uncommon, adverse effect of methadone [1]. It should be noted that the ToxIC Registry does not record minor prolongations of the QT interval. The criterion for QTc prolongation in our database is for it to be over 500 milliseconds. Thus, the actual number of cases that had less consequential QT prolongation was undoubtedly higher. However, complications such as torsade de pointes are unlikely at these lower QTc intervals. Nine and a half percent of our patients had acute kidney injury (AKI). Methadone-induced AKI, which may be a consequence of rhabdomyolysis, has been previously reported [23].

Interestingly, we found that 2% of patients experienced seizures. Most of these patients ingested a high dose of methadone. Methadone-induced seizure has been previously reported [24]. Few studies have evaluated the convulsive effects of methadone and the mechanism behind it. Animal studies showed that acute administration of methadone could substantially reduce the seizure threshold. NMDA and μ-opioid receptors may be involved in methadone’s convulsive activity in the acute methadone overdose [25].

In our study, it was found that just half of the patients with respiratory depression received naloxone. Similarly, Aghabiklooei et al. evaluated 322 serious pure methadone-poisoned patients. In their study, naloxone was administered for the treatment of respiratory depression to 40% of cases in the emergency department or during hospitalization [26]. As with any opioid poisoning, patients with respiratory depression or hypoxia require either naloxone administration or mechanical respiratory support [27].

### Limitations

The number of centers in the ToxIC Registry has changed over time. This is because the quality control procedures in ToxIC have caused poorly performing centers to be dropped, while new centers have joined the Consortium. The total number of cases reported each year has not varied widely, suggesting that the time trends we observed were not due to changes in the total number of cases reported to the ToxIC Registry. Further, as reviewed above, our time trends of serious methadone poisoning cases comport with those seen for all methadone poisonings in other national studies.

Secondly, we have reported the rates of consultations to medical toxicology services and not actual poisoning rates. Thus, our report likely represents the frequency of more serious cases of methadone toxicity. Because the treatment of methadone poisoning has not changed substantially over the study period, it is unlikely that the rate of consultation for serious cases could explain the decline starting in 2015. This suggests that professional and national efforts to curtail opioid toxicity have resulted in a trend of decreasing numbers of cases of serious methadone intoxication.

Understanding the pattern of opioid use in the US is necessary before effective measures to reduce morbidity and mortality from opioid use can be instituted. The opioid epidemic continued to increase after 2017; however, we could not present that data after 2018. Despite this, the time trends represent a component of the overall dynamic of “waves of the opioid epidemic.” By 2018, the “third wave” created by fentanyl and its analogs was underway, with methadone playing a lesser role.

## Conclusion

Our data demonstrate that rates of methadone poisoning increased in 2010–2014, followed by a decline in 2015–17.

## Supplementary information

**Additional file 1: Table 1.** Distribution of clinical effects based on the chronicity of use in methadone poisoning patients **Table 2.** Frequency of Receiving naloxone based on clinical effects

## Data Availability

The datasets used and/or analysed during the current study available from the corresponding author on reasonable request. All data used for this study was obtained from the Toxicology Investigators Consortium (https://www.ToxICRegistry.org) with permission by ToxIC after an application by the first author, who is currently in possession of the data set used in this analysis. Because the Western Institutional Review Board (WIRB) has concluded that the data collection in ToxIC does not meet the definition of Human Subjects Research and was approved by ToxIC no further ethics committee permission was deemed necessary.
