# Supplementary material for: Clinical characteristics and time trends of hospitalized methadone exposures in the United States based on the Toxicology Investigators Consortium (ToxIC) case registry: 2010–2017
Source: BMC Pharmacol Toxicol. 2020 Jul 22;21:53. doi: 10.1186/s40360-020-00435-0 (PMC7376634; doi:10.1186/s40360-020-00435-0)
Supplement: Supplementary file 1 — Additional file 1: Table 1. Distribution of clinical effects based on the chronicity of use in methadone poisoning patients Table 2. Frequency of Receiving naloxone based on clinical effects [file 40360_2020_435_MOESM1_ESM.docx]

Table 1: Distribution of clinical effects based on the chronicity of use in methadone poisoning patients

| Variable | Total | Chronic (n=60) | Acute on chronic (n=136) | Acute (n=411) |
| --- | --- | --- | --- | --- |
| Coma/CNS (%) | 472(48.6%) | 47(78.3%) | 65(47.8%) | 260(66.3%) |
| Agitation (%) | 69(7.1%) | 10(16.6%) | 17(12.5%) | 42(10.2%) |
| Seizures | 21(2.2%) | 2(3.33%) | 5(3.67%) | 14(3.40%) |
| Weakness | 6(0.6%) | 2(3.33%) | 0(0.0%) | 4(0.97%) |
| pH<7 (%) | 57(5.8%) | 2(3.33%) | 8(5.8%) | 47(11.4%) |
| Bradycardia (%) | 41(4.2%) | 4(6.66%) | 14(10.3%) | 23(5.59%) |
| Hypertension (%) | 34(3.5%) | 7(11.66%) | 18(13.2%) | 9(2.19%) |
| Hypotension (%) | 38(3.9%) | 1(1.66%) | 8(5.8%) | 29(7.05%) |
| Tachycardia (%) | 22(2.3%) | 4(6.66%) | 9(6.61%) | 9(2.19%) |
| QTc>500 milliseconds | 24(2.9%) | 4(6.66%) | 7(5.1%) | 13(3.16%) |
| Acute Kidney injury (%) | 92(9.5%) | 9(15.0%) | 26(19.1%) | 57(13.9%) |
| Rhabdomyolysis (%) | 43(4.4%) | 4(6.66%) | 11(8.1%) | 28(6.81%) |
| Aspiration pneumonitis | 53(5.4%) | 9(15.0%) | 14(10.3%) | 30(7.29%) |
| Respiratory depression | 327(33.6%) | 33(55.0%) | 91(66.9%) | 203(49.4%) |
| Hepatotoxicity (AST>1000) | 36(3.7%) | 3(5.0%) | 9(6.61%) | 24(5.84%) |
| The percentages in the methadone only and methadone plus coingestants groups are given for each group | | | | |

Table 2: Frequency of Receiving naloxone based on clinical effects

| Variable | Total | Naloxone | |
| --- | --- | --- | --- |
|  |  | Yes (n=442) | No (n=531) |
| Coma/CNS depression | 472(48.6%) | 308(69.8%) | 166(31.3%) |
| Agitation | 69(7.1%) | 25(5.66%) | 44(8.29%) |
| Seizures | 21(2.2%) | 6(1.35%) | 15(2.82%) |
| Weakness | 6(0.6%) | 2(0.45%) | 4(0.75%) |
| pH<7 | 57(5.8%) | 25(5.66%) | 32(6.01%) |
| Bradycardia | 41(4.2%) | 16(3.62%) | 25(4.71%) |
| Hypertension | 34(3.5%) | 13(2.94%) | 21(3.95%) |
| Hypotension | 38(3.9%) | 19(4.29%) | 19(3.58%) |
| Tachycardia | 22(2.3%) | 8(1.81%) | 14(2.63%) |
| QTc>500 milliseconds | 24(2.9%) | 11(2.49%) | 13(2.45%) |
| Acute Kidney injury | 92(9.5%) | 54(12.2%) | 39(7.34%) |
| Rhabdomyolysis | 43(4.4%) | 24(5.43%) | 19(3.58%) |
| Aspiration pneumonitis | 53(5.4%) | 33(7.43%) | 20(3.77%) |
| Respiratory depression | 327(33.6%) | 226(51.3%) | 101(19.0%) |
| Hepatotoxicity (AST>1000) | 36(3.7%) | 24(5.43%) | 12(2.25%) |
